# The cytotoxicity of malathion and essential oil of *Nepeta crispa* (lamiales: lamiaceae) against vertebrate and invertebrate cell lines

**DOI:** 10.11604/pamj.2019.33.285.18776

**Published:** 2019-08-06

**Authors:** Amirhossein Zahirnia, Mitra Boroomand, Hassan Nasirian, Sara Soleimani-Asl, Aref Salehzadeh, Dara Dastan

**Affiliations:** 1 Department of Medical Entomology, School of Medicine, Hamadan University of Medical Sciences, Hamadan, Iran; 2 Department of Medical Entomology and Vector Control, School of Public Health, Tehran University of Medical Sciences, Tehran, Iran; 3 Department of Anatomy, School of Medicine, Hamadan University of Medical Sciences, Hamadan, Iran; 4 Medicinal Plants and Natural Products Research Center, School of Pharmacy, Hamadan University of Medical Sciences, Hamadan, Iran

**Keywords:** *Nepeta crispa*, cytotoxicity, essential oil, invertebrate cell line, malathion, vertebrate cell line

## Abstract

**Introduction:**

Pesticides are used as essential tools to control vector-borne diseases and agricultural pests and maintain quality and quantity crop production. Scientists attempt to use derived plant natural products due to environmental safety and low mammalian toxicity. Therefore, the cytotoxicity of malathion and *Nepeta crispa* essential oil against vertebrate L929 and invertebrate Sf9 cell lines were investigated.

**Methods:**

About 2×10^3^ cells were placed into the wells of a 96-well plate experiments. Then appropriate concentrations of malathion and *N. crispa* essential oil added to the wells. The cells were allowed to grow for 3-5 days and estimated the cell numbers. Control cell wells contained only cells with DMSO. All treatments and controls repeated at least four replicates.

**Results:**

About 2×10^3^ cells were placed into the wells of a 96-well plate experiments. Then appropriate concentrations of malathion and *N. crispa* essential oil added to the wells. The cells were allowed to grow for 3-5 days and estimated the cell numbers. Control cell wells contained only cells with DMSO. All treatments and controls repeated at least four replicates.

**Conclusion:**

Plant essential oil not only had no negative effects but also had boosting effects on the L929 cell viability. *Nepeta crispa* essential oil had negative effects on the Sf9 cell viability with the differences that derived plant natural products containing environmentally friendly and readily biodegradable derivatives, hydrolyzing rapidly in nature and nearly having no destructive effects on mammals and environment.

## Introduction

At present, pesticides with increasingly global marketing are used as an essential tool to control of vector-borne diseases and agricultural pests and maintain quality and quantity crop production. They also play a key role in the prevention and control of infectious diseases such as malaria, dengue, and filariasis [1]. Despite their importance for public health, there is concern about pesticides potential side effects. Exposure to insecticides has severe effects on reproductive performance in vertebrates. It may cause increasing rates of cryptorchidism and hypospadias male genital congenital anomalies in human populations. Various types of insecticide exposure may be a risk factor for cancers such as leukemia or lymphoma. The other undesirable effects of pesticides are may be direct toxicity to users, environmental pollution, ozone depletion, pesticide residues, and toxicity to non-target organisms. There is the link between humans who are occupationally in contact with insecticides and muscle fatigue, neurological diseases and psychotic disorders [2-6]. Insecticide resistance in arthropods of vectors of diseases and agricultural pests to synthetic insecticides has been considered as a substantial problem of the vector and pest management programs [7-14]. With great concern about environmental problems and human health of synthetic pesticides, scientists have attempted to use natural products derived from plants that are considered as an appropriate option for vector and pest management due to containing environmentally friendly and readily biodegradable derivatives [6, 15, 16]. Essential oils are fugitive oil compounds that are secondary metabolites of plants. Essential oils hydrolyze rapidly in nature and have less destructive effects on the environment because of their environmental safety and low mammalian toxicity [6, 17]. Many studies have been done on insecticides effects of essential oils. Botanical insecticide compounds inhibit the activity of enzymes that are required to protect insects from oxidative stress, resistance to insecticides and other damage to insects [16, 18-20].

Many Iranian wild flowers have medicinal and insecticidal properties. Several studies have been conducted on Lamiaceae family due to their toxic effects on various insect species. The family of Lamiaceae have high diversity and distribution in flora of Iran containing 46 genera and 420 species and sub-species [21]. *Nepeta* is a genus of Lamiaceae family that has been spread in many parts of the world, including Asia, Europe and North Africa. About of 280 annual or perennial worldwide species of *Nepeta* genus, there are 79 species present in Iran with of 38 species of native to the country [22, 23]. In traditional medicine, *Nepeta* species are widely used as antispasmodic, anti-asthma, sedative, treatment of various digestive, neurological and respiratory diseases [24-26]. It's anti-viral, anti-inflammatory and antioxidant properties also have been reported [27-29]. Many studies have shown the insecticide properties of α-pinene, 1,8-cineol and α-terpineol as the main components of *Nepeta* genus species [30-32]. *Nepeta crispa* Willd. (Lamiales: Lamiaceae) is one of the most aromatic plants in Iran which is popular in Iranian traditional medicine, especially people of Hamadan province. *Nepeta crispa* is autochthonous of Hamadan climate having insecticidal activity, and antimicrobial and antifungal properties [33, 34]. Many studies have been conducted on the cytotoxic and insecticidal properties of *N. crispa*, but a simultaneous comparative study about the cytotoxicity of *N. crispa* against the cell lines of invertebrates and vertebrates would be of particular importance. Therefore in this study, we compared the cytotoxicity of *N. crispa* essential oil with malathion against vertebrate and invertebrate cell lines.

## Methods

**Providing of insecticide and plant materials:** liquid technical grade of malathion (95%) were purchased from India's Haramba Company. The aerial parts (foliage) of *Nepeta crispa* during their flowering stage were collected from Avicenna Medicinal Herbs Research Center, Hamadan Province of Iran in June 2017. The plant was confirmed by a voucher specimen (no. 72) in the Department of Pharmacognosy, School of Pharmacy, Hamadan University of Medical Sciences, Hamadan, Iran.

**Plant essential oil isolation:** a total of 1000 g powder of shade-dried aerial parts of *N. crispa* were subjected to hydrodistillation using a clevenger-type apparatus for 4h. The essential oil was dehydrated over anhydrous sodium sulphate and transferred into amber-colored vials to store in a refrigerator at 4ºC for further work.

### Providing and maintaining cell lines

*Invertebrate cell line:* Sf9 cell line which was derived from the ovary of *Spodoptera frugiperda* (Smith) (Lepidoptera: Noctuidae) was provided from National Cell Bank of Pasture Institute of Iran. Sf9 cell line is routinely cultured and maintained at 27ºC in 5ml of Grace's insect cell culture medium in 25cm^2^ culture flasks, enriched with 10% fetal bovine serum at Pasture Institute of Iran. The cell doubling time for this cell was found to be 18-24h under optimum conditions. Cells were sub-cultured every 3 days [35].

*Vertebrate cell line:* L929 vertebrate cell line which was derived from mouse fibroblast cells used for this study. It was provided from National Cell Bank of Pasture Institute of Iran. It was maintained at 37ºC in 3ml of DMEM Media (Gibco^®^) in 25cm^2^ culture flasks, enriched with 10% fetal bovine serum and buffered with 4% sodium bicarbonate in an atmosphere of 5% carbon dioxide. The doubling time for cultures was approximately 24h and the cell was sub-cultured every 6 day [35].

**Cell bioassay:** technical grade of malathion (95%) was dissolved with ethanol 96% to prepare the concentrations of 10^-10^, 10^-9^, 10^-8^, 10^-7^, 10^-6^, 10^-5^, 10^-4^ and 10^-3^ μg^-μL^ containing 0.000095, 0.00095, 0.0095, 0.095, 0.95, 9.5, 95 and 950 μg^-μL^, respectively. Herbal essential oil (0.1 mg) of *Nepeta crispa* was dissolved with 1ml of DMSO (dimethyl sulfoxide) due to hydrophobic properties and then diluted with sterilized distilled water to prepare the concentrations of 10^-10^, 10^-9^, 10^-8^, 10^-7^, 10^-6^, 10^-5^, 10^-4^ and 10^-3^ ng^-μL^ containing 0.00001, 0.0001, 0.001, 0.01, 0.1, 1, 10 and 100 ng^-μL^, respectively. To consider the cytotoxicity of malathion and *N. crispa* essential oil against L929 and Sf9 cell lines, about 2×10^3^ cells per 100 μl of culture medium were placed into the wells of a 96-well plate experiments of treatments and then appropriate concentrations of *N. crispa* essential oil and malathion added to the wells. We allowed the cells to grow for 3-5 days, and estimated the number of cells as described. Control cell wells contained only cells with 1 μl^-mL^ of DMSO. All treatment and control experiments repeated at least four replicates.

**Estimation of cell number:** the base method for cell estimation is the Mossman method, which uses 3-(4,5-dimethylthiazol-2-yl)-2,5-difenyltetrazolium bromide (MTT, tetrazolium, compound). MTT is a quantitative coloring for living cells and cell proliferation, and it's a known method for invitro cytotoxicity which measures the active metabolism of the cells. In this coloring solution, dehydrogenase enzyme reduced the MTT and produced blue formazan. The wells of 96-well plates containing L929 and Sf9 cell lines were incubated with 10 μ MTT for 3h at 36ºC and 27ºC, respectively. After the blue formazan and cells settled out and the supernatant was removed, 100 μ of DMSO was added to any well of 96-well plate, shaked for 15 minutes and then the absorbance of the solution read at 492 nm using ELISA reader [36].

**Statistical analysis:** IBM SPSS statistics data editor version 24 was used for any statistical analyses. Wilcoxon signed ranks-test was used for comparing cytotoxicity of malathion and essential oil of *Nepeta crispa* between control and treatments, and treatments against L929 and Sf9 cell lines. P < 0.05 was considered significant. The trends of malathion and *N. crispa* essential oil cytotoxicity against L929 and Sf9 cell lines was estimated by Microsoft Office Excel 2013. The trends were drawn by clicking on graph line distribution and selecting “add trendline” option using Nasirian and Salehzadeh (2017a, b; 2019a, b) style [11, 37-40].

**Statistical analysis:** IBM SPSS statistics data editor version 24 was used for any statistical analyses. Wilcoxon signed ranks-test was used for comparing cytotoxicity of malathion and essential oil of *Nepeta crispa* between control and treatments, and treatments against L929 and Sf9 cell lines. P < 0.05 was considered significant. The trends of malathion and *N. crispa* essential oil cytotoxicity against L929 and Sf9 cell lines was estimated by Microsoft Office Excel 2013. The trends were drawn by clicking on graph line distribution and selecting “add trendline” option using Nasirian and Salehzadeh (2017a, b; 2019a, b) style [11, 37-40].

## Results

**Figure 1:**
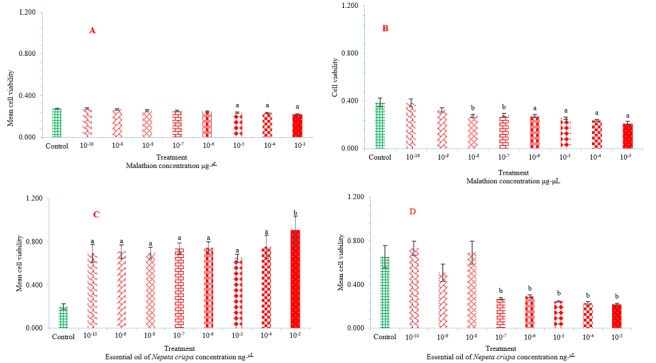
Cytotoxicity of malathion and essential oil of *Nepeta crispa* against L929 and Sf9 cell lines. A) Malathion against L929 cell lines; B) Malathion against Sf9 cell lines; C) Essential oil of *Nepeta crispa* against L929; D) Essential oil of *Nepeta crispa* against Sf9 cell lines. Sf9 cell line derived from the ovary of *Spodoptera frugiperda* (Smith) (lepidoptera: noctuidae). L929 vertebrate cell line derived from mouse fibroblast cells

**Figure 2:**
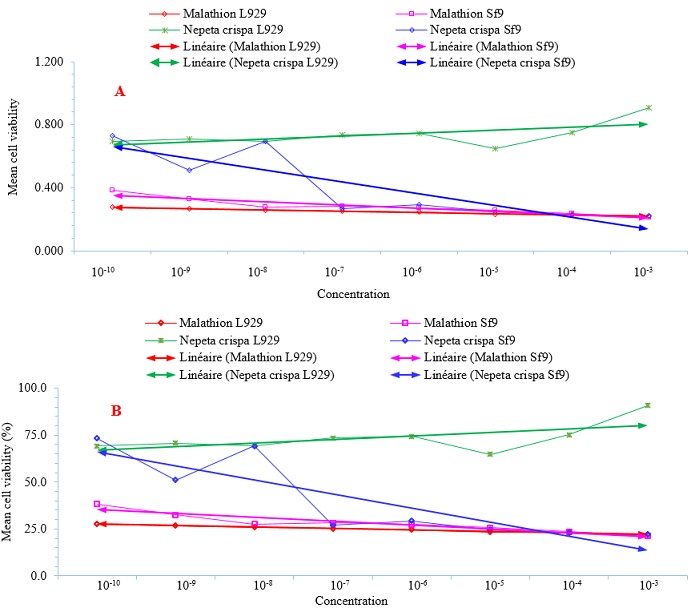
Cytotoxicity trends of malathion (μg^-μL^) and essential oil of *Nepeta crispa* (ng-^-μL^) against L929 and Sf9 cell lines. A) Normal data and B) Percent. The trends were drawn by clicking on graph line distribution and selecting “add trendline” option. Sf9 cell line derived from the ovary of *Spodoptera frugiperda* (Smith) (lepidoptera: noctuidae). L929 vertebrate cell line derived from mouse fibroblast cells

**Malathion cytotoxicity:** Table 1 and (Figure 1 A,B) show cytotoxicity of malathion and essential oil of *Nepeta crispa* (Figure 1 C,D) against L929 and Sf9 cell lines. Figure 2 also show the cytotoxicity trends of malathion (μg^-μL^) and essential oil of *Nepeta crispa* (ng^-μL^) against L929 and Sf9 cell lines. The cytotoxicity of malathion against L929 and Sf9 cell lines were gradually increased with a relatively low decreasing slope in accordance with malathion concentrations from 10^-10^ to 10^-3^ μg^-μL^ (Figure 1 A,B, Figure 2). Table 2 also shows the results of descriptive analysis and Wilcoxon signed-ranks test between control and treatments and between treatments of cytotoxicity of malathion and essential oil of *N. crispa* against L929 and Sf9 cell lines. There was a significant difference between treatments of 10^-5^ to 10^-3^ malathion concentrations against L929 cell lines with control (P < 0.05) (Figure 1 A and Table 2). There was also a significant difference between treatments of 10^-6^ to 10^-3^ malathion concentrations against Sf9 cell lines with control (P < 0.05) (Figure 1 B and Table 2). Although Wilcoxon signed-ranks test did not show a significant difference between treatments of 10^-8^ and 10^-7^ malathion concentrations against Sf9 cell lines with control (P > 0.05) (Table 2). While there was a significant difference at P < 0.001 level between treatments of 10^-8^ and 10^-7^ malathion concentrations against Sf9 cell lines with control (Figure 1 B).

**Table 1:** Cytotoxicity of malathion (μg-^μL^) and essential oil of *Nepeta crispa* (ng-^μL^) against L929 and Sf9 cell lines

C	R	Malathion		EONC		C	R	Malathion		EONC	
		L929	Sf9	L929	Sf9			L929	Sf9	L929	Sf9
		Control									
‒	R_1_	0.345	0.424	0.244	0.898	‒	R_5_	0.293	0.422	0.154	‒
‒	R_2_	0.296	0.384	0.276	0.731	‒	R_6_	0.273	0.342	0.104	‒
‒	R_3_	0.245	0.379	0.273	0.429	‒	R_7_	0.241	0.420	0.130	‒
‒	R_4_	0.263	0.177	0.265	0.564	‒	R_8_	0.235	0.555	0.159	‒
		**Treatment**									
10^-10^	R_1_	0.271	0.452	0.594	0.769	10^-6^	R_1_	0.249	0.327	0.578	0.279
	R_2_	0.301	0.334	0.556	0.561		R_2_	0.244	0.328	0.588	0.322
	R_3_	0.345	0.363	0.578	0.865		R_3_	0.276	0.262	0.583	0.301
	R_4_	0.288	0.357	0.278	0.732		R_4_	0.258	0.242	0.842	0.269
	R_5_	0.252	0.588	0.858	‒		R_5_	0.215	0.282	0.861	‒
	R_6_	0.237	0.321	0.862	‒		R_6_	0.247	0.270	0.893	‒
	R_7_	0.252	0.356	0.935	‒		R_7_	0.239	0.249	0.865	‒
	R_8_	0.262	0.293	0.873	‒		R_8_	0.237	0.229	‒	‒
10^-9^	R_1_	0.268	0.362	0.548	0.699	10^-5^	R_1_	0.238	0.293	0.598	0.242
	R_2_	0.309	0.327	0.525	0.340		R_2_	0.236	0.282	0.546	0.240
	R_3_	0.316	0.407	0.572	0.431		R_3_	0.256	0.243	0.870	0.262
	R_4_	0.243	0.346	0.538	0.569		R_4_	0.249	0.224	0.691	0.243
	R_5_	0.240	0.319	0.837	‒		R_5_	0.223	0.284	0.695	‒
	R_6_	0.247	0.280	0.867	‒		R_6_	0.221	0.233	‒	‒
	R_7_	0.249	0.285	0.876	‒		R_7_	0.231	0.256	‒	‒
	‒	‒	‒	‒	‒		R_8_	0.219	0.233	‒	‒
10^-8^	R_1_	0.260	0.336	0.554	0.692	10^-4^	R_1_	0.240	0.268	0.861	0.192
	R_2_	0.276	0.314	0.545	0.986		R_2_	0.233	0.233	0.909	0.217
	R_3_	0.298	0.238	0.538	0.593		R_3_	0.239	0.244	0.101	0.269
	R_4_	0.271	0.267	0.580	0.496		R_4_	0.245	0.259	0.878	0.235
	R_5_	0.239	0.304	0.813	‒		R_5_	0.219	0.242	0.723	‒
	R_6_	0.242	0.258	0.827	‒		R_6_	0.228	0.216	0.699	‒
	R_7_	0.244	0.250	0.865	‒		R_7_	0.221	0.204	0.707	‒
	R_8_	0.236	0.235	0.837	‒		‒	‒	‒	‒	‒
10^-7^	R_1_	0.253	0.330	0.560	0.239	10^-3^	R_1_	0.224	0.285	0.573	0.191
	R_2_	0.274	0.305	0.588	0.292		R_2_	0.229	0.239	0.546	0.235
	R_3_	0.277	0.299	0.575	0.268		R_3_	0.213	0.191	0.545	0.222
	R_4_	0.274	0.284	0.750	0.277		R_4_	0.220	0.115	0.668	0.231
	R_5_	0.231	0.313	0.830	‒		R_5_	0.216	0.223	‒	‒
	R_6_	0.231	0.240	0.859	‒		R_6_	0.224	0.212	‒	‒
	R_7_	0.245	0.250	0.872	‒		R_7_	0.217	0.214	‒	‒
	R_8_	0.238	0.232	0.854	‒		‒	‒	‒	‒	‒

C= Concentration, EONC=Essential oil of *Nepeta crispa* and R=Replicate. Sf9 cell line derived from the ovary of *Spodoptera frugiperda* (Smith) (Lepidoptera: Noctuidae). L929 vertebrate cell line derived from mouse fibroblast cells.

**Table 2:** Results of Wilcoxon signed-ranks test between cytotoxicity of malathion and essential oil of *Nepeta crispa* against L929 and Sf9 cell lines

Descriptive statistics									
Malathion					Essential oil of *Nepeta crispa*				
	Mean	Std. deviation	Mean	Std. deviation		Mean	Std. deviation	Mean	Std. deviation
	L929		Sf9			L929		Sf9	
Control	0.27388	0.036760	0.38788	0.105627	Control	0.20063	0.070888	0.65550	0.203454
Treatment					Treatment				
10^-10^	0.27600	0.034690	0.38300	0.094820	10^-10^	0.69175	0.227017	0.73175	0.126884
10^-9^	0.26743	0.032129	0.32575	0.045071	10^-9^	0.70800	0.174798	0.50975	0.157420
10^-8^	0.25825	0.022018	0.27525	0.037852	10^-8^	0.69488	0.151501	0.69175	0.211859
10^-7^	0.25288	0.019694	0.28156	0.036561	10^-7^	0.73600	0.139069	0.26900	0.022316
10^-6^	0.24563	0.017517	0.27363	0.037075	10^-6^	0.74429	0.151629	0.29275	0.023641
10^-5^	0.23413	0.013378	0.25600	0.026939	10^-5^	0.64713	0.103931	0.24675	0.010243
10^-4^	0.23214	0.009907	0.23588	0.021748	10^-4^	0.75221	0.300670	0.22825	0.032387
10^-3^	0.22043	0.005563	0.21100	0.047860	10^-3^	0.90650	0.363210	0.21975	0.019923
**Wilcoxon signed-ranks test**									
**Between control and treatments**									
**Malathion**						**Essential oil of *Nepeta crispa***			
		Mean ranks		Z	*P*-value (2-tailed)	Mean ranks		Z	*P*-value (2-tailed)
		Negative	Positive			Negative	Positive		
**L929**									
	10^-10^	6.0	3.6	0.0001^a^	1.00	0.001	4.5	-2.521^b^	0.012
	10^-9^	4.8	3.0	-0.845^c^	0.398	0.001	4.0	-2.366^b^	0.018
	10^-8^	6.0	3.0	-0.840^c^	0.401	0.001	4.5	-2.521^b^	0.012
	10^-7^	6.3	2.8	-0.980^c^	0.327	0.001	4.5	-2.521^b^	0.012
	10^-6^	4.9	3.3	-1.612^c^	0.107	0.001	4.0	-2.366^b^	0.018
	10^-5^	4.9	2.0	-2.240^c^	0.025	0.001	4.5	-2.521^b^	0.012
	10^-4^	4.0	0.001	-2.366^c^	0.018	1.0	4.5	-2.197^b^	0.028
	10^-3^	4.0	0.001	-2.366^c^	0.018	0.001	2.5	-1.826^b^	0.068
**Sf9**									
	10^-10^	4.0	5.3	-0.280^c^	0.779	2.0	3.0	-0.365^b^	0.715
	10^-9^	4.0	4.0	-1.016^c^	0.310	3.5	1.5	-0.730^c^	0.465
	10^-8^	4.6	4.0	-1.960^c^	0.050	2.0	3.0	-0.365^b^	0.715
	10^-7^	4.4	5.0	-1.820^c^	0.069	2.5	0.001	-1.826^c^	0.068
	10^-6^	4.9	2.0	-2.240^c^	0.025	2.5	0.001	-1.826^c^	0.068
	10^-5^	5.0	1.0	-2.380^c^	0.017	2.5	0.001	-1.826^c^	0.068
	10^-4^	4.5	1.0	-2.197^c^	0.028	2.5	0.001	-1.826^c^	0.068
	10^-3^	4.0	0.001	-2.366^c^	0.018	2.5	0.001	-1.826^c^	0.068
**Between treatments of malathion and essential oil of *Nepeta crispa***									
		**L929**				**Sf9**			
10^-10^	10^-10^	5.0	1.0	-2.380^c^	0.017	2.5	0.001	-1.826^c^	0.068
10^-9^	10^-9^	4.0	0.001	-2.366^c^	0.018	2.5	0.001	-1.826^c^	0.068
10^-8^	10^-8^	4.5	0.001	-2.521^c^	0.012	2.5	0.001	-1.826^c^	0.068
10^-7^	10^-7^	4.50	0.001	-2.521^c^	0.012	0.001	2.5	-1.826^b^	0.068
10^-6^	10^-6^	4.0	0.001	-2.371^c^	0.018	2.5	2.5	0.0001^a^	1.000
10^-5^	10^-5^	4.5	0.001	-2.521^c^	0.012	1.5	3.5	-0.736^b^	0.461
10^-4^	10^-4^	4.5	1.0	-2.197^c^	0.028	3.0	2.3	-0.730^b^	0.465
10^-3^	10^-3^	2.5	0.001	-1.826^c^	0.068	3.0	2.0	-0.365^c^	0.715

a The sum of negative ranks equals the sum of positive ranks

b based on negative ranks and

c based on positive ranks. The *P*-value of significant (*P* < 0.05) are shown in bold font style.

Sf9 cell line derived from the ovary of *Spodoptera frugiperda*(Smith) (Lepidoptera: Noctuidae). L929 vertebrate cell line derived from mouse fibroblast cells.

***Nepeta crispa* essential oil cytotoxicity:** the cytotoxicity of *Nepeta crispa* essential oil against L929 cell lines were gradually decreased with a moderately increasing slope in accordance with *N. crispa* essential oil concentrations from 10^-10^ to 10^-3^ μg-μL (Figure 1 C, Figure 2). While the cytotoxicity of *N. crispa* essential oil against Sf9 cell lines were strongly increased with an intensive increasing slope in accordance with *N. crispa* essential oil concentrations from 10^-10^ to 10^-3^ μg^-μL^ (Figure 1 D, Figure 2). Wilcoxon signed-ranks test revealed a significant difference between treatments of 10^-10^ to 10^-4^ *N. crispa* essential oil concentrations against L929 cell lines with control (P < 0.05) (Figure 1 C and Table 2). Although Wilcoxon signed-ranks test did not show a significant difference between treatments of 10^-3^ *N. crispa* essential oil concentration against L929 cell lines with control (P = 0.068) (Table 2). While there was a significant difference at P < 0.001 level between treatments of 10^-3^ *N. crispa* essential oil concentration against Sf9 cell lines with control (Figure 1 C). Wilcoxon signed-ranks test did not show a significant difference between treatments of 10^-10^ to 10^-3^ *N. crispa* essential oil concentrations against Sf9 cell lines with control (P > 0.05) (Figure 1 B and Table 2), even though there was a significant difference at P < 0.001 level between treatments of 10^-7^ and 10^-3^ of *N. crispa* essential oil concentrations against Sf9 cell lines (Figure 1 D).

**Comparison between cytotoxicity of malathion and *N. crispa* essential oil:** the cytotoxicity of malathion against L929 and Sf9 cell lines were gradually increased with a relatively low decreasing slope in accordance with malathion concentrations. The cytotoxicity of *N. crispa* essential oil against L929 cell lines were gradually decreased with a moderately increasing slope in accordance with *N. crispa* essential oil concentrations. While the cytotoxicity of *N. crispa* essential oil against Sf9 cell lines were strongly increased with an intensive increasing slope in accordance with *N. crispa* essential oil concentrations (Figure 2). Wilcoxon signed-ranks test revealed a significant difference between treatments of 10^-10^ to 10^-4^ malathion concentrations with *N. crispa* essential oil, respectively against L929 cell lines (P < 0.05) (Table 2). Even though there was a significant difference between treatments of 10^-3^ malathion concentration with *N. crispa* essential oil at P < 0.001 level. Although Wilcoxon signed-ranks test did not show a significant difference between treatments of 10^-10^ to 10^-3^ malathion concentrations with *N. crispa* essential oil against Sf9 cell lines (P > 0.05). While there was a significant difference at P < 0.001 level between treatments of 10^-10^ to 10^-7^ malathion concentrations with *N. crispa* essential oil against Sf9 cell lines (Table 2).

## Discussion

In recent years, insect insecticide resistance, ecosystem and food chain pesticide contamination, extinction of non-target organisms, mutation, and soil and water pollution has been critical problems. The scientists attempt to use natural products derived from plants. Based on the results of the study, malathion had negative effects on the viability of both L929 and Sf9 cell lines. This results confirm the reports of the previous studies that concluded insecticides decreased growth of the vertebrate and invertebrate cell lines [41, 42]. Compared with the experiments of malathion treatments, the highest rate of cell viability was observed in the control group which did not receive any toxic agent. While, the viability of cell lines which were exposed to the different concentrations of malathion was lower than control group and decreased with increasing malathion concentrations (Figure 1 A,B, Figure 2). The negative effects of malathion on the viability of both L929 and Sf9 cell lines were also confirmed by Wilcoxon signed-ranks test by observing a significant difference between treatments of 10^-5^ to 10^-3^ and 10^-8^ to 10^-3^ malathion concentrations with control group against the L929 and Sf9 cell lines, respectively at P < 0.05 or P < 0.001 levels (Figure 1 A,B) and Table 2.

Unlike malathion, essential oil of *Nepeta crispa* did not have negative effects on the viability of L929 cell lines. Compared with the control group, the highest rate of cell viability was observed in the experiment treatments which were treated with *N. crispa* essential oil. The viability of L929 cell lines which were exposed to different concentrations of *N. crispa* essential oil was higher than control group and increased with increasing *N. crispa* essential oil concentrations (Figure 1 C, Figure 2). The boosting effects of *N. crispa* essential oil on the viability of L929 cell lines were also confirmed by Wilcoxon signed-ranks test by observing a significant difference between treatments of 10^-10^ to 10^-3^ *N. crispa* essential oil concentrations with treatments of malathion 10^-10^ to 10^-3^ concentrations and control group against the L929 cell lines at P < 0.05 or P < 0.001 levels (Figure 1 C) and Table 2. In addition to some previous benefits of the natural products deriving from plants [32, 43], this boosting effects of *N. crispa* essential oil on the viability of vertebrate cell lines may be considered as the new benefits of the natural products deriving from plants.

Like malathion, essential oil of *N. crispa* had negative effects on the viability of Sf9 cell lines with the differences that the natural products deriving from plants containing environmentally friendly and readily biodegradable derivatives, hydrolyzing rapidly in nature and nearly having no destructive effects on mammals, humans or the environment [6, 15]. In addition, the application of *N. crispa* essential oil concentrations (ng^-μL^) was 1,000 folds lower than malathion concentrations (μg^-μL^). The viability of Sf9 cell lines which were exposed to concentrations of 10^-9^ and 10^-7^ to 10^-3^ *N. crispa* essential oil was lower than control group and decreased with increasing *N. crispa* essential oil concentrations (Figure 1 D, Figure 2). The negative effects of *N. crispa* essential oil on the viability of Sf9 cell lines were also confirmed by Wilcoxon signed-ranks test by observing a significant difference between treatments of 10^-7^ to 10^-3^ *N. crispa* essential oil concentrations with control group against the Sf9 cell lines at P < 0.001 level (Figure 1 D). But with application of the natural products deriving from plants, there is no concern about their potential side effects like direct toxicity to users, environmental pollution, ozone depletion, pesticide residues and toxicity to non-target organisms. We will not face up to severe insecticide effects on vertebrate reproductive performance, and encounter probably some type of cancers or muscle fatigue, neurological diseases and psychotic disorders [2-6]. Maybe no longer encounter to a substantial problem of the pest and vector management programs due to insecticide resistance in arthropods of the agricultural pests and vectors of diseases [7-14].

## Conclusion

Pesticides are used as an essential tool to vector-borne diseases and agricultural pests, and maintain quality and quantity crop production. With great concern about environmental problems and human health of synthetic pesticides, scientists have attempted to use natural products derived from plants. *Nepeta crispa* (Lamiales: Lamiaceae) is one of the most aromatic plants in Iran. *N. crispa* is autochthonous of Hamadan climate. A simultaneous comparative study about the cytotoxicity of *N. crispa* against the cell lines of invertebrates and vertebrates would be a particular of importance. Therefore, the cytotoxicity of *N. crispa* essential oil and malathion against L929 cell line of vertebrates and Sf9 cell line of invertebrates were investigated. Based on the results of the study, malathion had negative effects on the viability of both L929 and Sf9 cell lines. Unlike malathion, essential oil of *N. crispa* not only did not have negative effects on the viability of L929 cell lines, but also have boosting effects on the viability of L929 cell lines. Significant differences are also observed between treatments of 10^-10^ to 10^-3^ *N. crispa* essential oil concentrations with treatments of malathion 10^-10^ to 10^-3^ concentrations and control group against the L929 cell lines by Wilcoxon signed-ranks test confirming this fact. Like malathion, essential oil of *N. crispa* had negative effects on the viability of Sf9 cell lines with the differences that the natural products deriving from plants containing environmentally friendly and readily biodegradable derivatives, hydrolyzing rapidly in nature and nearly having no destructive effects on mammals, humans or the environment. In addition, the application of *N. crispa* essential oil concentrations was extremely lower than malathion concentrations. On the other hand there is no concern about plant essential oil potential side effects like direct toxicity to users, environmental pollution, ozone depletion, pesticide residues, and toxicity to non-target organisms with application of the natural products deriving from plants. We will not also encounter probably some type of cancers or muscle fatigue, neurological diseases, and psychotic disorders by applying the derived natural products of plants. As well as maybe no longer encounter to a substantial problem of the vector and pest management programs due to insecticide resistance in arthropods of the vectors of diseases and agricultural pests.

### What is known about this topic

- Scientists attempt to use derived plant natural products due to environmental safety and low mammalian toxicity;
- *Nepeta crispa* (lamiales: lamiaceae) is one of the most aromatic plants in Iran which is popular in Iranian traditional medicine.

### What this study adds

- Plant essential oil not only had no negative effects but also had boosting effects on the L929 cell viability.
- *Nepeta crispa* essential oil had negative effects on the Sf9 cell viability with the differences that derived plant natural products containing environmentally friendly and readily biodegradable derivatives, hydrolyzing rapidly in nature and nearly having no destructive effects on mammals and environment.

## Competing interests

The authors declare no competing interests.
